# Early-Season Host Switching in *Adelphocoris* spp. (Hemiptera: Miridae) of Differing Host Breadth

**DOI:** 10.1371/journal.pone.0059000

**Published:** 2013-03-19

**Authors:** Hongsheng Pan, Yanhui Lu, Kris A. G. Wyckhuys

**Affiliations:** 1 State Key Laboratory for Biology of Plant Diseases and Insect Pests, Institute of Plant Protection, Chinese Academy of Agricultural Sciences, Beijing, China; 2 International Center for Tropical Agriculture CIAT-Asia, Hanoi, Vietnam; BASF Cropdesign, Belgium

## Abstract

The mirid bugs *Adelphocoris suturalis* (Jakovlev), *Adelphocoris lineolatus* (Goeze) and *Adelphocoris fasciaticollis* (Reuter) (Hemiptera: Miridae) are common pests of several agricultural crops. These three species have vastly different geographical distributions, phenologies and abundances, all of which are linked to their reliance on local plants. Previous work has shown notable differences in *Adelphocoris* spp. host use for overwintering. In this study, we assessed the extent to which each of the *Adelphocoris* spp. relies on some of its major overwinter hosts for spring development. Over the course of four consecutive years (2009–2012), we conducted population surveys on 77 different plant species from 39 families. During the spring, *A. fasciaticollis* used the broadest range of hosts, as it was found on 35 plant species, followed by *A. suturalis* (15 species) and *A. lineolatus* (7 species). Abundances of the species greatly differed between host plants, with *A. fasciaticollis* reaching the highest abundance on Chinese date (*Ziziphus jujuba* Mill.), whereas both *A. suturalis* and *A. lineolatus* preferred alfalfa (*Medicago sativa* L.). The host breadths of the three *Adelphocoris* spp. differed greatly between subsequent spring and winter seasons. The generalist species exhibited the least host fidelity, with *A. suturalis* and *A. lineolatus* using 8 of 22 and 4 of 12 overwinter host species for spring development, respectively. By contrast, the comparative specialist *A. fasciaticollis* relied on 9 of its 11 overwinter plants as early-season hosts. We highlight important seasonal changes in host breadth and interspecific differences in the extent of host switching behavior between the winter and spring seasons. These findings benefit our understanding of the evolutionary interactions between mirid bugs and their host plants and can be used to guide early-season population management.

## Introduction

Host plant use forms the basis of niche breadth and the evolutionary success of herbivores [1]. Depending upon individual host breadth and other ecological particularities, herbivorous insects transfer between host plant species to differing extents to locate suitable host foods for their offspring and themselves [2], [3], [4], [5]. For generalist herbivores, a mixing of diets can produce substantial benefits, and a selective intake of food items or host plant species can redress or prevent nutritional imbalances [6], [7]. Subsequently, host switching can be employed as an adaptation to restricted food sources, and eventually result in improved fitness or subsequent population build-up [3], [8]. In addition to revealing key aspects of the evolution of plant-animal systems, a knowledge of host breadth and host switching behavior can help to understand the source-sink dynamics of agricultural pests.

In China, *Adelphocoris suturalis* (Jakovlev), *A. lineolatus* (Goeze) and *A. fasciaticollis* (Reuter) are three common pest species on cotton, alfalfa and many other crops [9], [10]. Both adults and nymphs feed on the vegetative and reproductive organs of their host plants, causing stunted growth and the abscission or malformation of leaves, flowers and fruits [11]. Over the past 15 years, an increased adoption of transgenic Bt (*Bacillus thuringiensis*) cotton and the subsequent reduction in insecticide use in this crop have increased *Adelphocoris* spp. infestation levels [12].

The three *Adelphocoris* spp. have different geographical distributions, seasonal occurrences and infestation levels. *A. suturalis* is mainly found in temperate areas, such as the Yangtze River Region and the southern part of the Yellow River Region, whereas *A. lineolatus* and *A. fasciaticollis* are usually confined to colder regions, namely certain parts of the Yellow River Region [10], [13]. Local agro-landscape composition and the phenology and abundance of suitable host plants are thought to determine *Adelphocoris* spp. population abundances in each of these regions [9], [14]. Each *Adelphocoris* species has a specific range of overwintering host plants that it uses, largely consistent with each species’ distribution and phenology [15].

On these winter hosts, the different *Adelphocoris* species overwinter as eggs. Some insect species rely on a wide range of plant species for overwintering, whereas others have a far more restricted host range. The eggs of *A. suturalis* have successfully eclosed from 115 plant species, whereas *A. lineolatus* and *A. fasciaticollis* have overwintered on 40 and 35 plant species, respectively [15]. The following spring, the overwintering eggs hatch, and newly emerged nymphs begin feeding on several plant species for one generation; then, the adults subsequently move onto summer host plants. The presence of suitable host plants in or near *Adelphocoris* spp. overwintering sites is particularly important given the limited dispersal capacities of the newly emerged nymphs [11]. It is unknown to what extent the different *Adelphocoris* spp. rely on overwinter hosts for spring development and whether early-season host use relates to the dietary breadth of a given species.

In this study, we contrasted the early-season host plant range of the three *Adelphocoris* species with previously reported winter host use patterns. The results may help explain interspecific differences in the distributions and phenologies of *Adelphocoris* spp. Additionally, a sound understanding of early-season host switching and population buildup could ultimately help predict *Adelphocoris* spp. infestation levels in summer crops such as cotton and alfalfa.

## Materials and Methods

### Ethics Statement

No specific permits were required for the described field studies.

### Field Trials

Field surveys were conducted from mid-April to mid-June of the year 2009–2012 at the natural areas and agricultural fields near the Langfang Experiment Station, Chinese Academy of Agricultural Sciences (CAAS) (116.4 °E, 39.3 °N), in Hebei Province, China. Here, all three *Adelphocoris* spp. have similarly low population levels [10], [15].

Each year, we sampled various plant species (including weeds, fruit trees, economic trees, pastures, and agricultural crops) that are common and widely distributed in the agroecosystems of northern China based on information from local plant guides. A total of 77 plant species from 39 families were sampled, including 53 weeds, 20 trees, 2 pasture crops and 2 agricultural crops. We sampled 65 plant species covering 10,790 m^2^ (in 2009), 67 species covering 11,769 m^2^ (2010), 43 species covering 8,417 m^2^ (2011) and 56 species covering 4,345 m^2^ (2012) (Tables 1 and 2).

**Table 1:** Weedy host plants of *Adelphocoris* spp. in the spring and the winter during 2009–2012 at Langfang, Hebei Province, China.

Plant species	Sampling area (m^2^)				*A. suturalis*		*A. lineolatus*		*A. fasciaticollis*	
	2009	2010	2011	2012	Winter	Spring	Winter	Spring	Winter	Spring
Amaranthaceae										
*** Amaranthus retroflexus*** ** L.**	16	93		42	+	−	+	−	−	−
Asclepiadaceae										
* Cynanchum chinense* R. Br.	65	8		1		−		−		−
* Cynanchum thesioides* (Freyn) K. Schum.	10	2				−		−		−
* Metaplexis japonica* (Thunb.) Makino		8	20	6		−		−		+
Boraginaceae										
* Bothriospermum chinense* Bge.	150	5	8	1		−		−		−
* Lycopsis orientalis* L.	2					−		−		−
Brassicaceae										
* Descurainia sophia* (Linn.) Webb ex Prantl	19	16	49			−		−		−
* Lepidium sativum* L.	110	53	83	4		+		+		+
Chenopodiaceae										
*** Chenopodium album*** ** L.**	284	115	15	5	+	+	+	−	−	+
*** Salsola collina*** ** Pall.**	424	239	285	20	+	+	−	+	+	+
*** Kochia scoparia*** ** (L.) Schrad.**	9	7			+	+	+	−	+	+
* Chenopodium glaucum* L.	1	4		8		−		−		+
* Chenopodium serotinum* L.	31	3	16	12		−		−		+
Compositae										
*** Artemisia argyi*** ** Levl. et Vant.**	12	4		10	+	−	−	−	+	+
*** Xanthium sibiricum*** ** Patrin ex Widder**	16	3		10	+	−	−	−	−	+
*** Artemisia annua*** ** L.**	90	4	23	24	+	−	+	−	−	+
*** Lactuca indica*** ** L.**	320	46	81		+	−	−	−	−	-
*** Artemisia lavandulaefolia*** ** DC. Prodr.**	41	7	40	40	+	−	+	−	−	+
*** Artemisia scoparia*** ** Waldst. et Kit.**	340	36	18	22	+	−	-	−	+	+
*** Taraxacum mongolicum*** ** Hand.-Mazz.**	4	6		9	+	−	+	−	−	+
* Bidens pilosa* L.		1				−		−		−
* Carduus crispus* L.	10					−		−		−
* Cephalanoplos setosum* (Willd.) Kitam.	1	1	24	8		−		−		+
* Cirsium setosum* (Willd.) MB.	103	159	30	8		+		+		+
* Comnyza canadensis* (L.) Cronq.			2			−		−		−
* Hemistepta lyrata* Bunge	72	9	5	1		−		−		−
* Heteropappus altaicus* (Willd.) Novopokr	20	7	15	12		−		−		+
* Inula japonica* Thunb.	9					−		−		−
* Sonchus oleraceus* L.	1	1		10		−		−		+
Convolvulaceae										
* Calystegia hederacea* Wall.	64	89		11		+		−		−
* Convolvulus arvensis* L.		3	26	51		−		−		+
Cruciferae										
* Capsella bursa-pastoris* (L.) Medic.	13	18	2	6		−		−		−
Equisetaceae										
* Equisctum ramosissimum* Desf.	5	3		1		−		−		−
Euphorbiaceae										
* Euphorbia esula* L.	1	1				−		−		−
Gramineae										
*** Setaria viridis*** ** (L.) Beauv.**	9	17		1	−	−	−	−	−	−
* Echinochloa crusgalli* (L.) Beauv.		3		6		−		−		−
* Imperata cylindrica* (L.) Beauv.	17	109	2			−		−		−
* Phragmites communis* Trin.	55	28		8		−		−		−
* Poa annua* L.	30			2		−		−		−
Labiatae										
* Lagopsis supina* (Steph.) Ik.-Gal. ex Knorr.	890	228	103	9		+		−		+
*** Leonurus sibiricus*** ** L.**	4	2		4	+	−	−	−	−	+
Lamiaceae										
* Salvia plebeia* R. Br.	11	3		9		−		−		+
Leguminosae										
* Gueldenstaedtia multiflora* Bunge.		7				−		−		−
Malvaceae										
*** Abutilon theophrasti*** ** Medic.**	10	13	1	20	+	−	−	−	−	+
Moraceae										
*** Humulus scandens*** ** (Lour.) Merr.**	109	182	153	42	+	+	+	+	+	+
Plantaginaceae										
* Plantago depressa* Willd.	28	18	24	2		−		−		+
Polygonaceae										
* Polygonum aviculare* L.	14	10	1			−		−		−
Portulacaceae										
* Portulaca oleracea* L.	1	8		25		−		−		−
Rosaceae										
* Potentilla supina* L.	1	1		1		−		−		−
Rubiaceae										
* Rubia cordifolia* L.	17	15	3	9		−		−		+
Scrophulariaceae										
* Rehmannia glutinosa* Libosch.	17	25		14		−		−		+
Umbelliferae										
* Cnidium monnieri* (L.) Cuss.	1	2		3		+		−		−
Violaceae										
* Viola prionantha* Bunge.	1	1				−		−		−

Sampling area refers to the combined area covered by each sampled plant species in the respective year. The information of overwinter host ranges of *Adelphocoris* spp. is cited from [15]. The signs “+” and “−” indicate that the associated plant species is a host plant or non-host plant, respectively. A blank space indicates that this species was not surveyed. Plant species highlighted in bold were included in the host plant surveys during the winter [15] and the spring (present study).

**Table 2:** Cultivated host plants of *Adelphocoris* spp. in the spring and the winter during 2009–2012 at Langfang, Hebei Province, China.

Plant species	Sampling area (m2)				*A. suturalis*		*A. lineolatus*		*A. fasciaticollis*	
	2009	2010	2011	2012	Winter	Spring	Winter	Spring	Winter	Spring
Fruit tree										
Begoniaceae										
* Begonia grandis* Dry.			1			−		−		−
Ebenaceae										
* Diospyros kaki* Thunb.			3	265		−		−		−
Moraceae										
* Morus alba* L.	254	1876	293	61		−		−		+
Juglandaceae										
* Juglans regia* L.			1	136		−		−		−
Rhamnaceae										
*** Ziziphus jujuba*** ** Mill.**	1060	1048	670	562	+	−	+	+	+	+
Rosaceae										
*** Pyrus bretschneideri*** ** Rehd.**	1437	2422	1318	593	+	−	−	−	+	+
*** Malus domestica*** ** Borkh.**			3	69	+	−	+	−	+	−
*** Prunus persica*** ** (L.) Batsch**	2169	1930	1574	591	+	+	−	−	+	−
*** Prunus armeniaca*** ** L.**	964	1648	876	335	+	+	−	−	+	+
* Cerasus pseudocerasus* (Lindl.) G. Don		28								
* Crataegus pinnatifida* Bge.	16	74		82		−		−		+
* Prunus salicina* Lindl.	623	504	1103	413		−		−		−
Vitaceae										
*** Vitis vinifera*** ** L.**	422	254	730	284	+	+	+	−	+	+
Economic tree										
Leguminosae										
*** Amorpha fruticosa*** ** L.**	27	4		67	−	+	−	−	−	−
* Robinia pseudoacacia* L.	21	11	44			−		−		−
Rutaceae										
* Zanthoxylum bungeanum* Maxim.			3	127		−		−		−
Salicaceae										
*** Salix matsudana*** ** Koidz.**	18		25	53	−	−	−	−	−	−
*** Populus tomentosa*** ** Carr.**	40	16	694	99	−	−	−	−	−	−
Simaroubaceae										
* Ailanthus altissima* Swingle	55	1				−		−		−
Ulmaceae										
*** Ulmus pumila*** ** L.**	59	43	18	81	−	−	−	−	−	+
Pasture										
Leguminosae										
*** Melilotus suaveolens*** ** Ledeb.**	10	11	3		+	−	+	+	−	+
*** Medicago sativa*** ** L.**	81	50			+	+	+	+	−	+
Agricultural crop										
Agrostidoideae										
* Triticum aestivum* L.	74	225	29	50		+		−		−
Liliaceae										
* Allium fistulosum* L.	2	1				−		−		−

Sampling area refers to the combined area covered by each sampled plant species in the respective year. The information of overwinter host ranges of *Adelphocoris* spp. is cited from [15]. The signs “+” and “−” indicate that the associated plant species is a host plant or non-host plant, respectively. A blank space indicates that this plant was not surveyed. Plant species highlighted in bold were included in the host plant surveys during the winter [15] and the spring (present study).

The sampling protocol was adapted from an existing one [16]. In brief, the *Adelphocoris* spp. abundance on different plants was assessed using a standard white pan beating method. For herbaceous plants, we examined the entire plant; whereas for tree crops, we sampled the young branches. Sampling was performed every 3–5 days, the plant material was shaken over a 40 cm×26 cm×11 cm white pan, and the dislodged *Adelphocoris* individuals (adults and nymphs) were counted [17]. Identification of *Adelphocoris* species was based on morphological features [18]. Per year, a total of 10–16 sampling events were conducted, with 10–20 random samples taken per plant species and event. For common plant species, a single sample consisted of a total area of 2–20 m^2^, whereas for uncommon species, all of the plants at a given site were sampled. At each event, we determined the exact area covered by each plant species (i.e., sampling area) and recorded the plant growth stage. To correctly identify the associations of a particular *Adelphocoris* sp. with a given plant species, we only selected uniform patches or carefully chose single stems of a given plant species for sampling. Plant species were identified using regional weed guides [19] or with the assistance of CAAS plant taxonomists. Plant species on which individuals of each *Adelphocoris* sp. were found were defined as ‘host plants’ of the respective species [16], [20], and those host plants that had a wide distribution and supported high densities of *Adelphocoris* sp. were regarded as the species’ ‘dominant hosts’ [16].

### Statistical Analysis

For each *Adelphocoris* sp., the average abundance on each plant species was computed on a yearly basis, i.e., by dividing the total number of captured individuals on one plant species by the total area covered by this respective plant throughout the entire sampling period [16]. As the field survey generally started before overwintering eggs had begun to hatch, survey data were not included in the analyses before the appearance of the first individuals of *Adelphocoris* spp. The abundance of each *Adelphocoris* sp. was compared between different dominant plant species using a two-way un-replicated ANOVA with a Friedman's test, with years and plant species as fixed factors. A Chi-square test was performed to compare the rate at which overwinter host plants were also used as spring hosts between the three *Adelphocoris* species. All of the statistical analyses were performed using SAS software [21].

## Results

**Figure 1:**
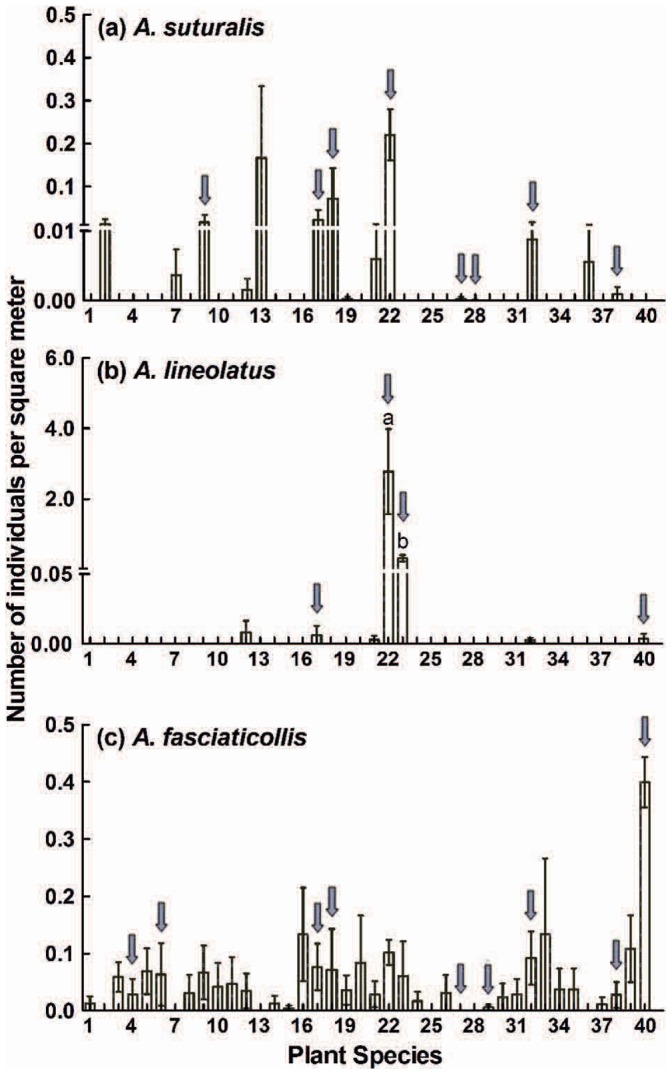
Comparison of the population density of each *Adelphocoris* species on different plant species. Data are shown as mean ± SE. Different letters denote significant differences between plant species. The gray arrows indicate that the plant species are both overwinter and spring hosts for a specific *Adelphocoris* sp. Plant species: 1 *Abutilon theophrasti* Medic., 2 *Amorpha fruticosa* L., 3 *Artemisia annua* L., 4 *Artemisia argyi* Levl. et Vant., 5 *Artemisia lavandulaefolia* DC. Prodr., 6 *Artemisia scoparia* Waldst. et Kit., 7 *Calystegia hederacea* Wall., 8 *Cephalanoplos setosum* (Willd.) Kitam., 9 *Chenopodium album* L., 10 *Chenopodium glaucum* L., 11 *Chenopodium serotinum* L., 12 *Cirsium setosum* (Willd.) MB., 13 *Cnidium monnieri* (L.) Cuss., 14 *Convolvulus arvensis* L., 15 *Crataegus pinnatifida* Bge., 16 *Heteropappus altaicus* (Willd.) Novopokr., 17 *Humulus scandens* (Lour.) Merr., 18 *Kochia scoparia* (L.) Schrad., 19 *Lagopsis supina* (Steph.) Ik.-Gal. ex Knorr., 20 *Leonurus sibiricus* L., 21 *Lepidium sativum* L., 22 *Medicago sativa* L., 23 *Melilotus suaveolens* Ledeb., 24 *Metaplexis japonica* (Thunb.) Makino, 25 *Morus alba* L., 26 *Plantago depressa* Willd., 27 *Prunus armeniaca* L., 28 *Prunus persica* (L.) Batsch, 29 *Pyrus bretschneideri* Rehd., 30 *Rehmannia glutinosa* Libosch., 31 *Rubia cordifolia* L., 32 *Salsola collina* Pall., 33 *Sonchus oleraceus* L., 34 *Salvia plebeia* R. Br., 35 *Taraxacum mongolicum* Hand.-Mazz., 36 *Triticum aestivum* L., 37 *Ulmus pumila* L., 38 *Vitis vinifera* L., 39 *Xanthium sibiricum* Patrin ex Widder, 40 *Ziziphus jujuba* Mill.

For *A. suturalis*, 15 species of host plants were found in the spring (Tables 1 and 2), but no significant difference was found for its population abundance on any of the host species (*X^2^* = 9.21, df = 14, *P = *0.8176). From the analyses of plant distribution and *A. suturalis* abundance on these 15 plant species, alfalfa *Medicago sativa* L. (0.22 individuals per m^2^), and four weeds *Cnidium monnieri* (L.) Cuss. (0.17), *Kochia scoparia* (L.) Schrad (0.07), *Humulus scandens* (Lour.) Merr. (0.02), and *Chenopodium album* L. (0.02) were regarded as the major spring host plants. From a total of 22 *A. suturalis* overwinter hosts, 8 species were confirmed as spring host plants, including *C. album*, *H. scandens*, *K. scoparia*, *M. sativa*, *Prunus armeniaca* L., *Prunus persica* (L.) Batsch, *Salsola collina* Pall., and *Vitis vinifera* L. (Figure 1a).

For *A. lineolatus*, 7 species of spring host plants were found (Tables 1 and 2). On alfalfa, *M. sativa*, the average abundance of *A. lineolatus* was 2.77±1.21 individuals per m^2^, which was significantly higher than on any other plant (*X^2^* = 13.16, df = 4, *P = *0.0405). The second highest abundance was 0.34 individuals per m^2^ on another pasture crop *Melilotus suaveolens* Ledeb, and those on all of the other host species were less than 0.01. From a total of 12 overwinter host plants, 4 species (incl. the above two pasture crops, and *H. scandens*, *Ziziphus jujuba* Mill.) were found to be *A. lineolatus*’s spring host plants (Figure 1b).

For *A. fasciaticollis*, 35 species of early-season host plants were found (Table 1 and 2), with no significant difference in population abundance (*X^2^* = 42.33, df = 34, *P = *0.1545). Chinese date, *Z. jujuba*, was considered a key spring host plant because of its large growing area and the high abundance of *A. fasciaticollis* (0.40±0.04 individuals per m^2^), and the population abundance on 4 host species, including *Morus alba* L., *P. armeniaca*, *Crataegus pinnatifida* Bge., and *Pyrus bretschneideri* Rehd, was less than 0.01. Among 11 *A. fasciaticollis* winter hosts, 9 species were regarded as its spring hosts, including *Artemisia argyi* Levl. et Vant., *Artemisia scoparia* Waldst. et Kit., *H. scandens*, *K. scoparia*, *P. armeniaca*, *P. bretschneideri*, *S. collina*, *V. vinifera*, and *Z. jujuba* (Figure 1c).

During the spring, the outspoken generalist *A. suturalis* and *A. lineolatus* were found on 36.4% (8/22) and 33.3% (4/12) of their overwinter plants. However, for *A. fasciaticollis*, 81.8% (9/11) of overwinter plants were also used as early-season hosts. The extent of using overwinter plants as early-season hosts significantly differed between the three *Adelphocoris* spp. (*X^2^* = 7.26, df = 2, *P = *0.0267). Additionally, 5 plant species, including *Cirsium setosum* (Willd.) MB., *H. scandens*, *Lepidium sativum* L., *M. sativa*, and *S. collina*, were shared as early-season host plants by all three mirid bug species (Table 3).

**Table 3:** Host fidelity of *Adelphocoris* spp. between the winter and the spring.

Mirid bug species	Feeding types	No. host plants^a^	No. overwinter hosts^b^	No. spring hosts^c^	No. spring hosts/No. overwinter hosts^d^	Host fidelity
*Adelphocoris suturalis*	Generalist	270	115	15	8/22	Low
*Adelphocoris lineolatus*	Generalist	245	40	7	4/12	Low
*Adelphocoris fasciaticollis*	Comparative specialist	127	35	35	9/11	High

a The total number of host plants includes the previously recorded hosts [11], the recently found winter hosts [15], the recently found spring hosts (present study), and the recently found summer hosts (Lu YH, Unpublished data).

b The overwinter host range is reported by [15].

c Number of spring hosts determined in the present study.

d Number of overwinter hosts [15] used as spring hosts in this study.

## Discussion

For *Adelphocoris* spp., early-season host plants are the key source for future colonization or the exploitation of summer hosts such as cotton. To date, the host plant ranges of various mirid bugs (e.g., *Lygus rugulipennis* Poppius, *Lygus lineolaris* (Palisot de Beauvois), *Lygus hesperus* Knight, *Apolygus lucorum* (Meyer-Dür)) in the spring have been determined [16], [20], [22], [23], [24], [25]. This survey determined that there are 15 species of early-season host plants for *A. suturalis*, 7 species for *A. lineolatus*, and 35 species for *A. fasciaticollis* in northern China. Several early-season host plants had been previously identified for these *Adelphocoris* spp. in China [9], [14], [26], [27], [28]. However, because these studies were conducted at different locations with differing species compositions and differing abundances of *Adelphocoris* spp. and plants, their results cannot be used to explore between-species differences in distribution and seasonal occurrence. Our present study effectively complements previous work because all three *Adelphocoris* spp. coexist at similar population levels at the study site [15].

In 2008, literature reviews and exploratory host range trials indicated that there was a total of 116, 125 and 30 host plant species for *A. suturalis*, *A. lineolatus* and *A. fasciaticollis*, respectively [11]. Novel work brought the respective host plant range of *A. suturalis*, *A. lineolatus* and *A. fasciaticollis* to 270, 245 and 127 species, maintaining the previous interspecific differences in host breadth (Lu YH, unpublished data; Table 3). Because of the limited abundance/cover at sampling sites, certain plant species were only sampled in 1–2 m^2^ in this study. Although limited sampling might lead to underestimates of the host range of a given *Adelphocoris* spp., plant species with low abundance/cover in natural and agricultural habitats will only play a minor role in the population dynamics of the different mirid bugs. Hence, the updated results presented here provide a comprehensive set of information on year-round host plant range for future research on the interactions between *Adelphocoris* spp. and its host plants and the regional management of these polyphagous pests.

Stark differences were found between the host breadth of the three *Adelphocoris* spp. during the winter and spring season. For *A. suturalis*, a limited set of host plants was found during the spring compared to their overall host range and overwinter host range of 270 and 115 species, respectively (Table 3). In the Yangtze River region, where *A. suturalis* is dominant, several important host plants such as horse bean (*Vicia faba* L.), carrot (*Daucus carota* L.), garland chrysanthemum (*Chrysanthemum coronarium* L.), celery (*Apium graveolens* L.), alfalfa and hairy vetch (*Vicia villosa* Roth), are cultivated to a large extent [9], [27]. The fact that the above host plants are grown to a lesser extent in northern China may partly explain the relatively low population levels of this pest locally.

For *A. lineolatus*, alfalfa was the principal early-season host plant and is also an important overwinter host for this species [15]. Large areas of alfalfa, cultivated as a pasture crop, could explain the relatively high population levels of *A. lineolatus* in one of China’s key cattle growing areas (i.e., Cangzhou, Hebei Province) [11], [29]. Indeed, *A. lineolatus* adults greatly prefer alfalfa to other host plants [29], but periodic rotation of alfalfa fields can cause adults to disperse to cotton, sunflower and other crops. As new alfalfa fields are established, *A. lineolatus* adults gradually migrate back to the alfalfa fields [29]. These phenomena indicate that alfalfa is the most important host plant for *A. lineolatus*, which greatly affects its distribution and phenology.

For *A. fasciaticollis*, the early-season host range was similarly as broad as the overwinter host range, with 35 plant species reported as overwinter hosts [15]. Chinese date was the most important overwinter and early-season host plant. It was previously thought that trees were significant hosts for *A. fasciaticollis*, but because no individuals were found on other fruit trees, such as *P. persica* and *Malus domestica* Borkh., the *A. fasciaticollis* life cycle may be mainly restricted to Chinese date [11], [26]. Chinese date is primarily grown in northern China [30], which could explain why *A. fasciaticollis* is mainly confined to this part of the country [10], [18].

During the host-plant selection process of phytophagous insects, the successful colonization of suitable host plants is pivotal for their individual survival and population build-up. For specialist insect species, it may be more difficult and dangerous to change food plants and seek a new host than for generalists [31]. Hence, in general, the degree of host fidelity of comparative specialists tends to be higher than for generalists [32]. In our study, the different *Adelphocoris* species exhibited varying levels of fidelity to their overwinter host plants, with the (comparative) specialist *A. fasciaticollis* exhibiting the greatest extent of host fidelity. This finding supports the above general viewpoint on host fidelity of phytophagous insects.

Host fidelity does not necessarily imply increased survival because host switching can cause additional mortality. Even for species that use overwinter host plants for spring development, survival rates can be as low as 30% [33]. For species such as *A. suturalis* and *A. lineolatus,* that use an entirely new set of plants for early-season development, host switching could constitute an additional mortality factor [34]. Consequently, it is expected that host switching leads to a fitness increase that effectively compensates for this additional mortality. In addition to host plant ranges, the fitness of *Adelphocoris* spp. on different hosts can thus help explain between-population differences in many life-history traits.

Our work shows large seasonal variability in host usage patterns. For *A. suturalis* and *A. lineolatus*, a relatively small set of host plants was recorded during the spring compared to their overall host range, which comprises 270 and 245 species, respectively (Table 3). *A. fasciaticollis* adopted a fairly similar host range in spring and winter seasons but exhibited the broadest host range in the spring season, being a comparative specialist. Seasonal differences in host usage likely relate to the nutritional profile of a given plant species for (spring) nymphal development versus physical attributes that provide shelter for winter eggs. Nevertheless, the large differences in host plant ranges of both *A. suturalis* and *A. lineolatus* between subsequent seasons must be further analyzed. More precisely, the relationship between (autumn) adult oviposition preference and offspring performance merits further study [35], [36]. As both of the populations appear to experience a ‘bottleneck’ in the spring, important opportunities for population management could be identified [37].

Because *Adelphocoris* spp. complete their first generation on early-season host plants, these plants act as important sources for subsequent infestation of cotton and other summer agricultural crops. Hence, strategic management of early-season host plants could lead to important reductions of those summer populations. For instance, in the United State, broadleaf weeds are the main early-season host plants of the tarnished plant bug *L. lineolaris* before its movement into cotton fields [38]. Systematic removal of stands of broadleaf weeds near cotton plantings effectively reduced subsequent *L. lineolaris* numbers in cotton fields [39], [40]. Our work provides the basis for similar tactics for the suppression of early-season populations of *Adelphocoris* spp. in cotton agroecosystems in China.

## References

[1] Singer MC (1983) Determinants of multiple host use by a phytophagous insect population. Evolution 37: 389–403. 28568358 10.1111/j.1558-5646.1983.tb05547.x

[2] Velasco LRI, Walter GH (1993) Potential of host-switching in *Nezara viridula* (Hemiptera: Pentatomidae) to enhance survival and reproduction. Environ Entomol 22: 326–333.

[3] Panizzi AR (1997) Wild hosts of pentatomids: ecological significance and role in their pest status on crops. Annu Rev Entomol 42: 99–122. 15012309 10.1146/annurev.ento.42.1.99

[4] Wheeler AG Jr (2001) Biology of the plant bugs (Hemiptera: Miridae): pests, predators opportunists. New York: Cornell University Press. 507p.

[5] Coyle DR, Clark KE, Raffa KF, Johnson SN (2011) Prior host feeding experience influences ovipositional but not feeding preference in a polyphagous insect herbivore. Entomol Exp Appl 138: 137–145.

[6] Bernays EA, Minkenberg OPJM (1997) Insect herbivores: different reasons for being a generalist. Ecology 78: 1157–1169.

[7] Raubenheimer D, Jones SA (2006) Nutritional imbalance in an extreme generalist omnivore: tolerance and recovery through complementary food selection. Anim Behav 6: 1253–1262.

[8] Rossi AM, Brodbeck BV, Strong DR (1996) Response of xylem-feeding leafhopper to host plant species and plant quality. J Chem Ecol 22: 653–671. 24227575 10.1007/BF02033576

[9] Cao CY, Wan CS (1983) Management of cotton mirids. Shanghai: Shanghai Science and Technology Press. 60p.

[10] Lu YH, Qiu F, Feng HQ, Li HB, Yang ZC, et al (2008) Species composition and seasonal abundance of pestiferous plant bugs (Hemiptera: Miridae) on Bt cotton in China. Crop Prot 27: 465–472.

[11] Lu YH, Wu KM (2008) Biology and control of cotton mirids. Beijing: Golden Shield Press, 151p.

[12] Lu YH, Wu KM, Jiang YY, Xia B, Li P, et al (2010) Mirid bug outbreaks in multiple crops correlated with wide-scale adoption of Bt cotton in China. Science 328: 1151–1154. 20466880 10.1126/science.1187881

[13] Zhang SM, Zhao YX (1996) The geographical distribution of agricultural and forest insects in China. Beijing: China Agriculture Press, 304p.

[14] Gao ZR, Li QS, Qiu F, Wu YQ, Jiang DZ (1992) Studies on the Rb-marked cotton plant bugs and dispersal from weeds to cotton fields. Sci Agri Sin 25: 15–21.

[15] Lu YH, Jiao ZB, Li GP, Wyckhuys KAG, Wu KM (2011) Comparative overwintering host range of three *Adelphocoris* species (Hemiptera: Miridae) in northern China. Crop Prot 30: 1455–1460.

[16] Lu YH, Jiao ZB, Wu KM (2012) Early-season host plants of *Apolygus lucorum* (Heteroptera: Miridae) in northern China. J Econ Entomol 105: 1603–1611. 23156156 10.1603/ec12003

[17] Lu YH, Wu KM, Wyckhuys KAG, Guo YY (2010) Overwintering hosts of *Apolygus lucorum* (Hemiptera: Miridae) in northern China. Crop Prot 29: 1026–1033.

[18] Zheng LY, Lv N, Liu GQ, Xu BH (2004) Fauna Sinica, Insecta Vol. 33 (Hemiptera: Miridae: Mirinae). Beijing: Science Press, 797p.

[19] Wang ZR (1990) Farmland weeds in China: a collection of colored illustrative plates. Beijing: Agricultural Publishing House, 506p.

[20] Esquivel JF, Mowery SV (2007) Host plants of the tarnished plant bug (Heteroptera: Miridae) in central Texas. Environ Entomol 36: 725–730. 17716463 10.1603/0046-225x(2007)36[725:hpottp]2.0.co;2

[21] SAS Institute (2005) SAS/STAT user’s guide, version 9.13. Cary, NC, USA: SAS Institute.

[22] Scott DR (1977) An annotated listing of host plants of *Lygus hesperus* Knight. Bull Entomol Soc Amer 23: 19–22.

[23] Snodgrass GL, Scott WP, Smith JW (1984) An annotated list of the host plants of *Lygus lineolaris* (Hemiptera : Miridae) in the Arkansas, Louisiana, and Mississippi delta. J Georgia Entomol Soc 19: 93–101.

[24] Holopainen JK, Varis AL (1991) Host plants of the European tarnished plant bug *Lygus rugulipennis* Poppius (Het., Miridae). J Appl Entomol 111: 484–498.

[25] Robbins JT, Snodgrass GL, Harris FA (2000) A review of wild host plants and their management for control of the tarnished plant bug in cotton in the Southern U.S. Southwest Entomol. 23 Suppl 21–25.

[26] Chu HF, Meng HL (1958) Studies on three species of cotton plant bugs, *Adelphocoris taeniophorus* Reuter, *A. lineolatus* (Goeze), and *Lygus lucorum* Meyer-Dür (Hemiptera, Miridae). Acta Entomol Sin 8: 97–118.

[27] Liu HM (1991) Studies on the bionomics and control strategy of black-striped leaf bug in northern coastal reclamation area of Jiangsu province. Acta Phytophyl Sin 18: 147–153.

[28] Gao ZR, Li QS (1998) On the selectivity and dispersion of alfalfa plant bug among its host plants in eastern Henan cotton region. Acta Phytophyl Sin 25: 330–336.

[29] Li YF, Dang ZH, Gao ZL, Guo ZG, Xu JM, et al.. (2011) Primary study of seasonal occurrence of mirid bugs in mixed planting system of alfalfa and cotton. In Wu KM, editors. Thesis compilation of annual congress of Chinese Society of Plant Protection in 2011. Beijing: Chinese Agricultural Science and Technology Press. 210–212.

[30] Liu MJ (2008) China jujube development report (1949–2007). Beijing: Forestry Press of China, 193p.

[31] Müller J, Stadler J, Jarzabek-Müller A, Hacker H, ter Braak C, et al (2011) The predictability of phytophagous insect communities: host specialists as habitat specialists. PLoS ONE 6: e25986 doi:10.1371/journal.pone.0025986. 22016796 10.1371/journal.pone.0025986 PMC3189246

[32] Bernays EA (2001) Neural limitations in phytophagous insects: implications for diet breadth and evolution of host affiliation. Annu Rev Entomol 46: 703–727. 11112184 10.1146/annurev.ento.46.1.703

[33] Welsman JA, Bahlai CA, Sears MK, Schaafsma AW (2007) Decline of soybean aphid (Homoptera: Aphididae) egg populations from autumn to spring on the primary host, *Rhamnus cathartica* . Environ Entomol 36: 541–548. 17540062 10.1603/0046-225x(2007)36[541:dosaha]2.0.co;2

[34] Stoyenoff JL, Witter JA, Montgomery ME, Chilcote CA (1994) Effects of host switching on gypsy moth *(Lymantria dispar* (L.)) under field conditions. Oecologia 97: 143–157. 28313923 10.1007/BF00323144

[35] Thompson JN (1988) Evolutionary ecology of the relationship between oviposition preference and performance of offspring in phytophagous insects. Entomol Exp Appl 47: 3–14.

[36] Gripenberg S, Mayhew PJ, Parnell M, Roslin T (2010) A meta-analysis of preference-performance relationships in phytophagous insects. Ecol Lett 13: 383–393. 20100245 10.1111/j.1461-0248.2009.01433.x

[37] Kennedy GG, Storer NP (2000) Life system of polyphagous arthropod pests in temporally unstable cropping systems. Annu Rev Entomol 45: 467–493. 10761586 10.1146/annurev.ento.45.1.467

[38] Snodgrass GL, Scott WP, Smith JW (1984) Host plants and seasonal distribution of the tarnished plant bug (Heteroptera: Miridae) in the delta of Arkansas, Louisiana, and Mississippi. Environ Entomol 13: 110–116.

[39] Snodgrass GL, Scott WP, Abel CA, Robbins JT, Gore J, et al (2005) Tarnished plant bug (Heteroptera: Miridae) populations near fields after early season herbicide treatment. Environ Entomol 34: 705–711.

[40] Snodgrass GL, Scott WP, Abel CA, Robbins JT, Gore J, et al (2006) Suppression of tarnished plant bugs (Heteroptera: Miridae) in cotton by control of early season wild host plants with herbicides. Environ Entomol 35: 1417–1422.

